# Developing decision support tools for high-risk women and healthcare providers to increase chemoprevention informed choice and uptake: A retrospective translational science case study

**DOI:** 10.1017/cts.2024.565

**Published:** 2024-09-10

**Authors:** Leah G. Pope, Zainab Abedin, Katherine D. Crew, Rita Kukafka, Harold Alan Pincus

**Affiliations:** 1 Irving Institute for Clinical and Translational Research, Columbia University Irving Medical Center, New York, NY, USA; 2 Department of Psychiatry, Vagelos College of Physicians and Surgeons, Columbia University Irving Medical Center, New York, NY, USA; 3 New York State Psychiatric Institute, New York, NY, USA; 4 Department of Medicine, Vagelos College of Physicians and Surgeons, Columbia University Irving Medical Center, New York, NY, USA; 5 Department of Epidemiology, Mailman School of Public Health, Columbia University Irving Medical Center, New York, NY, USA; 6 Herbert Irving Comprehensive Cancer Center, Columbia University Medical Center, New York, NY, USA; 7 Department of Biomedical Informatics, Vagelos College of Physicians and Surgeons, Columbia University Irving Medical Center, New York, NY, USA; 8 Department of Sociomedical Sciences, Mailman School of Public Health, Columbia University Irving Medical Center, New York, NY, USA

## Abstract

Retrospective case studies are one approach to help identify processes underlying the translation of successful health interventions. This case study investigates the development of *RealRisks* and *Breast Cancer Risk Navigation* (*BNAV*), decision support tools for breast cancer risk assessment, and risk-stratified prevention. Following a recently developed protocol for retrospective translational science case studies, we examined the career trajectory of Dr Katherine Crew as she expanded from basic science to interdisciplinary, patient-oriented research in oncology and began collaboration with Dr Rita Kukafka, a public health informatician focused on communicating risk. Data collection methods included key informant interviews and examination of peer-reviewed publications, funded grants, and news articles associated with the research. Data were analyzed to identify key milestones in the development of *RealRisks* and *BNAV* and to elucidate facilitators and barriers to the translational process. Facilitators to translation included funding and infrastructure provided by a Clinical and Translational Science Award (CTSA), the creation of an interdisciplinary team, and broad support from stakeholders including patient advocacy groups. Barriers to translation included limited mid-career support, ongoing costs for technology, and the time required to establish interdisciplinary, team science efforts. The findings reported here can be used to inform ongoing efforts to develop a more robust science of translation.

## Introduction

Recent scholarship and federal funding opportunities have focused on the importance of investing in “translational science” initiatives that can help elucidate the scientific and operational principles underlying each step in the translational process [1–3]. Recognizing the inherent complexity of translation from scientific potential to medical reality, such translational science initiatives seek to better identify and understand the barriers in translational research and develop strategies and test hypotheses for overcoming those barriers. One method for elucidating the science of translation is to conduct case studies that evaluate the translational processes underlying the development of successful health interventions. Case studies are often used in evaluation research and can be especially valuable in the health field for describing how certain activities advance science and improve public health outcomes. Drawing on a recently developed protocol for case studies in translational science [4], we conducted a retrospective case study about the development of *RealRisks* and *Breast Cancer Risk Navigation* (*BNAV*), decision support tools for breast cancer risk assessment, and risk-stratified prevention for patients and healthcare providers, respectively. We highlight key milestones in the development of *RealRisks* and *BNAV*, elucidate the facilitators and barriers during the translational process, and describe the current state of dissemination and implementation. The objective of this case study is to contribute to generalizable insights about the contexts that drive translation forward.

## Breast cancer, chemoprevention, and informed patient choice

Breast cancer is the most commonly diagnosed cancer among women in the United States and imparts significant morbidity and mortality, with over 40,000 deaths annually [5]. It is estimated that at least 15% of women, ages 35–75 years old, in the U.S., are considered high-risk for breast cancer and may be eligible for chemoprevention [6]. Several randomized controlled trials (RCTs) provide evidence that chemoprevention agents (e.g., selective estrogen receptor modulators, such as tamoxifen, and aromatase inhibitors) reduce breast cancer incidence by up to 40%–65% for high-risk women [7]. This evidence of reduced risk through chemoprevention has led the U.S. Preventive Services Task Force to recommend clinicians offer to prescribe risk-reducing medications to women who are at increased risk for breast cancer and low risk for adverse medication effects [8]. Nonetheless, chemoprevention uptake among women in the U.S. is remarkably low. One systematic review found that the mean uptake rate of chemoprevention was less than 15% among women at high risk for breast cancer (and averaged only 4% when excluding one study that reported an uptake rate of more than 50%) [9]. Several studies have reported on the reasons for such low uptake, including concerns about side effects, limited physician knowledge, and the lack of tools or time to efficiently assess breast cancer risk in primary care settings [7,9]. Other research has demonstrated lower awareness and less uptake of chemoprevention among racial and ethnic minority women, differences that can lead to increased health disparities [10,11].

The barriers to chemoprevention uptake suggest that new strategies and tools are needed to identify high-risk women and inform them about the risks and benefits of chemoprevention. More specifically, tools must be developed that not only communicate risk in understandable and acceptable ways to patients but also give clinical providers the information and tools they need to facilitate timely discussions in primary care settings. It is in this context that a multidisciplinary team of researchers at Columbia University Irving Medical Center led by Drs. Katherine Crew and Rita Kukafka developed decision support tools for patients and providers known as *RealRisks* and *BNAV*, respectively. *RealRisks* and *BNAV* take a precision medicine approach to cancer prevention by quantifying personalized risk for breast cancer, communicating that risk to patients and providers, and improving the quality of shared decision-making around chemoprevention uptake.

## Case study methods

This case study was developed following Dodson and colleague’s protocol for retrospective case studies [4]. This includes steps to (1) review existing background information; (2) create an initial timeline with key milestones; (3) identify gaps in knowledge; (4) identify a list of key stakeholders; (5) conduct semi-structured interviews with key stakeholders; (6) continue gathering data until gaps in knowledge are addressed; (7) review findings with key stakeholders to ensure accuracy; and (8) finalize the case study and timeline.

We first reviewed background information, including searching PubMed for relevant peer-reviewed publications, identifying grants funded by the National Institutes of Health (NIH) through the NIH RePORTER database (2 R01s and 1 R21), and examining a prior interview and 15 peer-reviewed articles that had been written about the tools and associated research. Our team then developed an initial draft timeline with key milestones and assessed knowledge gaps. We used this information to develop a semi-structured interview protocol, utilizing the interview format outlined in Dodson et al [4]. After consulting with Dr Crew, we decided to interview her, her interdisciplinary research partner, Dr Rita Kukafka, and two of Dr Crew’s mentors who were instrumental to early career development and continue to be collaborators. Semi-structured interviews were conducted between September and November 2020 and lasted 30–60 minutes. Interviews were recorded and transcribed for analysis. A timeline of key events was created and reviewed with Drs. Crew and Kukafka. Follow-up interviews with Drs. Crew and Kukafka were held in Spring/Summer 2023 to gather updated data on progress of the research and resolve outstanding questions. Interviews were coded by the first two authors using a deductive approach with predetermined codes [12] identified in Dodson et al.’s (2021) protocol, including key events, progress milestones, key people and partnerships, other major facilitators and barriers (e.g., funding, technologies), impacts, and future directions. Findings were summarized following the approach described in Dodson et al.’s protocol [4].

## Case study findings

### Key events in the development of RealRisks and BNAV

**Figure 1.**
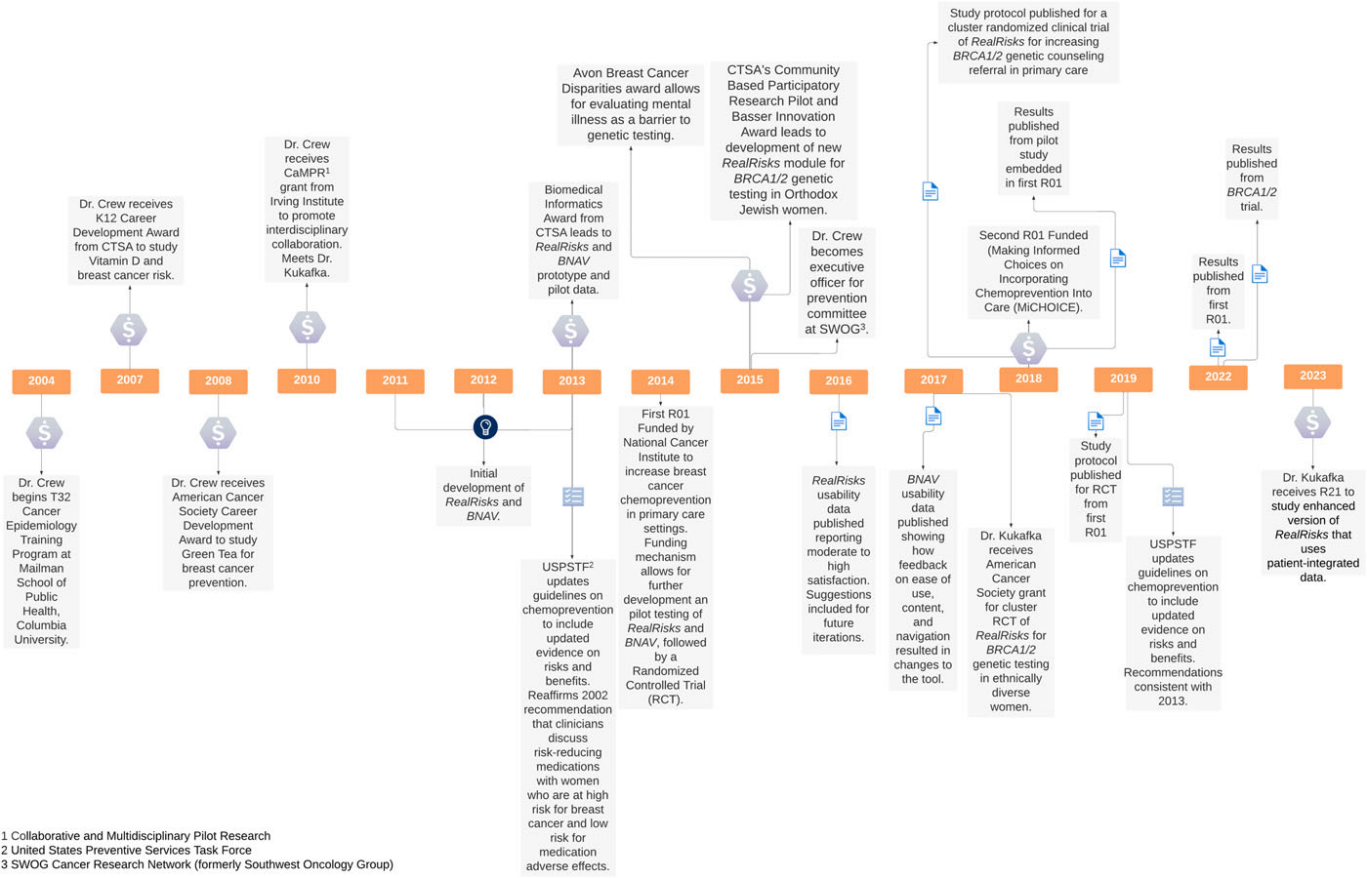
Timeline of key events in development of *RealRisks* and *BNAV*.

The development of *RealRisks and BNAV* is best understood through an examination of Dr Crew’s career progression and building of an interdisciplinary team with Dr Rita Kukafka (see Figure 1 for a timeline of key milestones in translation). Dr Crew began her faculty career in hematology/oncology and received several career development awards that provided a foundation for her early work as a physician-scientist. These included a K12 career development award from the Irving Institute for Clinical and Translational Research, a Career Development Award from the American Society of Clinical Oncology, and a Mentored Research Scholar Award from the American Cancer Society. Having done primarily laboratory-based research in molecular epidemiology during her fellowship training, these awards allowed Dr Crew to extend her observational research on novel agents for breast cancer prevention, such as Vitamin D and Green Tea, into patient-oriented research and clinical trials [13,14].

In 2010, as Dr Crew was in the process of transitioning from mentored research to independent research, she received a pilot award from the Irving Institute as part of its Collaborative and Multidisciplinary Pilot Research program (CaMPR) that would prove to be a critical turning point. The CaMPR program provides funds for new collaborations (e.g., for investigators who have not previously worked together). Dr Crew’s CaMPR award served as a planning grant for developing the Breast Cancer Family-Based Intervention Trial to target women with a first-degree family history of breast cancer in clinic and community-based settings and provide information and education about personal risk. In considering the best way to communicate personal risk of breast cancer, one of Dr Crew’s collaborators on the CaMPR award introduced Dr Crew to Dr Rita Kukafka, who specializes in risk communication and the development of clinical informatics tools.

Dr Kukafka had been working on interactive risk communication and building decision support tools before meeting Dr Crew but was focused on cardiovascular disease. As someone who considers herself “disease agnostic” in her research, Dr Kukafka found the underutilization of chemoprevention to be an interesting problem to collaborate on solving. Drs. Crew and Kukafka began to consider how to adapt some of the same strategies that Dr Kukafka was using for women at high risk for breast cancer. They decided to partner to develop a decision support tool for breast cancer prevention. Dr Crew would provide the expertise in breast cancer risk, prevention, and treatment; Dr Kukafka would provide expertise in building the architecture and user interface of the decision support tool that communicates risk and supports informed decision-making.

Dr Crew and Dr Kukafka’s collaboration led to a one-year Biomedical Informatics grant from the CTSA to develop a decision support tool for chemoprevention and collect pilot data. The result was the creation of *RealRisks* and *BNAV*. *RealRisks* and *BNAV* are web-based decision support tools for communicating breast cancer risk, reducing inaccurate risk perceptions, and providing preference-based decision support for managing risk. *RealRisks* is a patient-centered, web-based decision aid that calculates a women’s risk for developing breast cancer, determines her eligibility for chemoprevention, provides tailored education based on her risk profile, and elicits patient preferences and values surrounding breast cancer prevention decisions. Information is presented to patients about a fictitious character, Rose, in narrative and comic form and patients can interact with embedded games about breast cancer risk and prevention options. *BNAV* is the companion provider-centered decision support tool that summarizes patient risk profiles and preferences and provides educational resources to support provider decision-making around breast cancer risk reduction and facilitate patient-provider communication [7]. *RealRisks* and *BNAV* target both patients and healthcare providers at the same time, embed the *BNAV* report in an existing electronic health record, and target women in multiple clinical settings as well as from multiple different racial/ethnic backgrounds. The tool is updated annually to ensure it communicates the most up-to-date medical information to patients and providers. Initial pilot data demonstrated significant improvement in accuracy of breast cancer risk perception after interacting with *RealRisks* and also yielded qualitative data to inform the iterative design of the tool [15].

In 2014, Drs. Crew and Kukafka received NIH funding to conduct a pilot trial of *RealRisks* and *BNAV* in a breast clinic, followed by a RCT in a primary care setting [7,16]. The pilot trial enrolled 40 women. Accurate breast cancer risk perceptions increased from baseline to 6 months and chemoprevention knowledge significantly improved from baseline to post-intervention follow-up. However, there was no significant change in breast cancer knowledge, and decision conflict increased from post-intervention to 6 months [16]. Results informed the design and conduct of the larger RCT. The RCT targeted younger, healthier women with higher breast cancer risk who are likely to derive greater benefit from breast cancer risk reduction; it enrolled 282 high-risk women from a racially and ethnically diverse patient population, along with 45 providers. Results demonstrated that although *RealRisks* did not lead to increased chemoprevention uptake, it was associated with significant improvements in accurate breast cancer risk perceptions, adequate chemoprevention knowledge, and decision quality (including reduced decision conflict and increased informed choice) [17].

The first RCT fostered continued development of *RealRisks* and BNAV in two key ways. First, Drs. Crew and Kukafka were motivated to enhance the tools in new directions with additional risk stratification modules. In particular, they built out *RealRisks* to screen women for hereditary breast and ovarian cancer syndrome attributable to *BRCA1* and *BRCA2 (BRCA 1/2)* variants. This led to additional pilot funding from the Irving Institute and the Basser Center to expand work on use of the decision aid among Orthodox Jewish women eligible for *BRCA1/2* testing and evaluate mental illness as a barrier to genetic testing [18–20]. This work identified additional sociocultural and psychological barriers to uptake of genetic testing for hereditary breast cancer. Then, in 2017, Dr Kukafka received funding from the American Cancer Society for a cluster randomized clinical trial of this new module in ethnically diverse women [21,22]. Recently reported results showed that genetic counseling uptake within 6 months did not differ significantly between the intervention and control groups (*RealRisks* v. standard patient education). However, participants in the intervention group reported significantly greater improvements in genetic testing knowledge at 1 month and postclinic visit, favorable genetic testing attitudes at postclinic visit, and decreased breast cancer worry at postclinic visit and 6 months [22].

Second, to continue building the evidence base for chemoprevention uptake and to broaden generalizability, Drs. Crew and Kukafka sought to collaborate with the SWOG Cancer Research Network (formerly Southwest Oncology Group) to scale up and test *RealRisks* and *BNAV* in a multisite trial. The SWOG Cancer Research Network is one of the National Cancer Institute-funded cooperative groups that conducts multi-center, large-scale, cancer clinical trials. Dr Crew joined SWOG in 2005, facilitating partnership on a grant. Together, they received funding from the NIH in 2018 for a multisite trial among women with atypical hyperplasia or lobular carcinoma in situ. In comparison to the modules tested in the first RCT, where patients and providers accessed the tools through a link to an external website, the trial underway also integrates *RealRisks* and *BNAV* into the clinic workflow via electronic health records (EHR) and evaluates the impact of portal integration. The primary outcome measure is chemoprevention informed choice (rather than chemoprevention uptake as in the first RCT), reflecting the most updated research about the primary benefit of decision aids [23] as well as current guidelines from the U.S. Preventive Services Task Force that recommend clinicians discuss risk-reducing medications to women at increased risk for breast cancer and at low risk for adverse medication effects and participate in shared, informed decision-making [8].

### Facilitators of translation

#### Obtaining CTSA pilot funding

Dr Crew described the pilot funding she received from the Irving Institute – funding specifically targeted to promoting collaborative and multidisciplinary research – as being the most instrumental for the development of *RealRisks* and *BNAV*. Specifically, this funding was critical for enabling Dr Crew to develop new, interdisciplinary partnerships – including her partnership with Dr Kukafka – and in pushing the bounds of her research to include new methods (e.g., community-based participatory research) and new populations (e.g., Ashkenazi Jewish women). These initial pilot awards facilitated data collection to show the initial promise of the intervention/research program and apply for subsequent grant funding. The result is an interdisciplinary team, consisting of oncologists, epidemiologists, software developers, data scientists, and public health experts.

#### Interdisciplinary collaborations

Interview participants pointed to the centrality of building strong, interdisciplinary relationships to catalyze the development of clinical interventions and research programs. Although creating interdisciplinary teams requires patience, there was a recognition that the development of *RealRisks* and *BNAV* would not have been possible without robust collaboration between scientists from multiple disciplines working toward a common goal. Drs. Crew and Kukafka both reflected their sense that solving complex health problems requires a team approach, with Dr Kukafka reflecting, “health conditions don’t have a single etiology and neither do solutions.” They both felt their work was enriched by cross-fertilizing their thinking with people from other disciplines but noted that engaging in this type of work is partly a matter of personal interest (e.g.., according to Dr Kukafka, “I think there are some people who are inherently interested in [interdisciplinary work] and some people who are not”) and partly a result of infrastructures that support such interdisciplinary collaborations. In this respect, the development of *RealRisks* and *BNAV* was facilitated not only by funding to support interdisciplinary collaboration, but also by forums created by the Irving Institute and Columbia University Irving Medical Center in which researchers could come together from different areas of specialty, engage in opportunities for dialog, share ideas, and identify potential partnerships [24]. For example, Drs. Crew and Kukafka participated in and presented at the Columbia University Herbert Irving Comprehensive Cancer Center’s Interdisciplinary Research Series seminar in January 2021. This focus on interdisciplinarity at the institutional level aligns with vast literature supporting the importance of interdisciplinary work in promoting creativity and novel scientific discoveries [25–27].

Building relationships with outside researchers and groups was also critical. Dr Crew became involved in the SWOG Cancer Research Network in 2005 as a junior faculty member through one of her mentors. Over time, she continued to develop her connections to SWOG and take on leadership positions, becoming an executive officer for SWOG’s prevention committee in 2015. This proved vital for future research and SWOG is now the primary collaborator on the current multisite trial.

#### Support from patient advocacy groups

Patient advocates are a standard part of review committees, and reaching out to patient advocacy groups in designing and implementing *RealRisks* was critical for its development. Drs. Crew and Kukafka conducted usability studies of their tools with actual end users prior to launch to ensure that the interventions were feasible to use and acceptable to patients of different backgrounds [16]. Dr Crew noted that even as a clinician who has direct care responsibility for patients, talking to trained patient advocates provided a new avenue for understanding issues that patients may not want to share with their clinicians as well as for anticipating how the intervention would be received at a local and national level.

### Barriers to translation

#### Limited mid-career support

Keeping research funded is an ongoing challenge for scientists but can be particularly difficult for mid-career faculty [28]. While Dr Crew benefitted from several career development awards that informed her evolution as a cancer researcher, her work to develop *RealRisks* and *BNAV* came alongside her transition from mentored research to independent research. This meant she had less protected career development time and relatively fewer funding opportunities for the development and testing of the decision tool. In turn, Dr Crew became involved in multiple collaborations that could provide salary support – broadening the network for potential future collaborations but also reducing the amount of time available to focus on *RealRisks* and *BNAV* as it relates to chemoprevention.

#### Ongoing costs for technology

A separate issue – also related to funding – is that a web-based decision support tool requires ongoing financial investment, including funding to maintain secure servers and provide security updates. Drs. Crew and Kukafka have been able to maintain continuous support for *RealRisks* and *BNAV* by having multiple funders and expanding the reach of the tool beyond its initial focus on chemoprevention. However, given the precariousness of relying on federal funding – and the risk that a lapse in funding would mean shutting down the decision tools – they have started to explore other options for the future including potential paths to commercialization.

#### Establishing interdisciplinary, team science efforts

The development of *RealRisks* and *BNAV* required the collaboration across multiple disciplines. As one interviewee reflected, “If you’re trying to do something new or novel, you need people with a lot of different expertise, disease-based expertise, population science expertise, expertise in implementation science, and expertise in biomedical informatics. You need the whole spectrum, especially when you’re talking about developing new interventions and testing new interventions.” While such team science efforts are recognized as critical, they also present logistical and conceptual challenges. Not only are team members involved in multiple projects and being pulled in different directions, but their diverse backgrounds and areas of expertise can also make it hard to find a common language. Further, current promotion criteria do not always reward team science efforts, which can be a barrier to finding collaborators. Finding other scientists who are, as Kukafka described in her interview, “working at the edge of their discipline” requires patience and willingness. However, Drs. Crew and Kukafka have found that as their work has gained more visibility, other researchers who are interested in interdisciplinary work have sought them out for collaboration. By bringing in different domain experts to fill gaps in knowledge and respecting the expertise of each team member, they continue to expand the network of potential research partners with whom they can collaborate.

#### Integrating decision tools into clinical workflow

A final challenge in the translation of *RealRisks* and *BNAV* has been the integration of the tools at the practice level. As Crew and Kukafka’s team has documented in their qualitative work during tool development, providers face competing demands on their time during any clinical encounter and must establish priorities based on their patients’ needs. Further, primary care providers lack knowledge about chemoprevention and seldom engage in risk communication with their patients (particularly in time-constrained settings) [29]. Dr Kukafka explained how it can be challenging to “introduce prevention into a health care system that is more episodic and designed to manage acute conditions… it’s not a small challenge – how does it fit into the clinical work flow?” Responding to this challenge and initial results about uptake of *BNAV* among providers, the research team has since integrated *BNAV* into the EHR and is studying this integration in the RCT underway.

#### Current status and future directions

With the current multisite RCT of *RealRisks* and *BNAV* almost completed, Dr Crew and Kukafka’s team will soon have more information about the effectiveness of the decision tools in increasing chemoprevention-informed choice as well as their impact on secondary outcomes including chemoprevention knowledge, perceived breast cancer risk/worry, decision conflict, shared decision-making, and chemoprevention uptake and adherence. Results have the potential to inform widespread dissemination of the tool to improve informed, shared decision-making about breast cancer chemoprevention in line with current guidelines.

In the meantime, Drs. Crew and Kukafka have continued their work to add, adapt, and test new modules and enhancements for *RealRisks* and *BNAV*. Recent enhancements focus on patient activation. With the current integration of the tool into the EHR, *RealRisks* can automatically populate information for a breast cancer risk calculation and a new prototype interface shows this data to patients for review and modification. Patients can then add additional personal health information for running updated risk models and aligning each patient’s risk with appropriate preventive interventions. This enhanced version of *RealRisks* is now the subject of an R21 grant from the National Institute on Minority Health and Health Disparities that aims to conduct user evaluations; assess its effect on patient activation, risk perception, and usability among multi-ethnic high-risk women; and identify multilevel barriers to clinic implementation. The team hopes to empower patients to make deliberate decisions that are appropriate to their risk, make informed decisions, and feel activated in doing so.

Given the modular architecture of *RealRisks*, the team’s longer-term goal is to continue building it out as a personalized medicine tool that delivers risk-appropriate screening and prevention options depending on the individual user. This has relevance for diseases beyond breast and ovarian cancers. For example, given that many women at high risk for breast cancer are also at high risk for cardiovascular disease, the team has applied for a grant to study the impact of collecting cardiovascular disease risk through *RealRisks* and how women understand (and juxtapose) their cancer risk and cardiovascular disease risk. In this way, the team continues to enhance *RealRisks* to be applicable to an expanding number of health conditions that involve preference-sensitive decisions. They also continue to pursue pragmatic studies in real-world clinical practice to facilitate further integration of *RealRisks* and *BNAV* into clinic workflow and messaging.

## Conclusion

This case study on *RealRisks* and *BNAV* highlights key facilitators and barriers to successful translation. There are limitations to a case study approach. Like Dodson et al.’s protocol [4], we focus on central elements of discovery rather than the full scope of the research program, which may filter out important aspects. Our data is also drawn from the two lead scientists in this research and two early career mentors, so they are susceptible to subjective interpretation. Beyond these limitations, this specific case may be especially valuable for strategizing translational research initiatives that involve early career investigators, the development and integration of technology into clinic workflows, and the focus on a disease that has a strong patient advocacy community. Further, the facilitators identified by this case study are applicable to other health problems and research settings. The support and infrastructure provided by a CTSA, the creation of an interdisciplinary team, and broad support from stakeholders including patient advocacy groups contributed to successful research and translation of findings and can be harnessed in other research contexts. Similarly, the barriers faced by the research team provide important clues about common pitfalls other researchers may face in the process of translation and point to strategies for overcoming them. By highlighting these broadly applicable facilitators and barriers, case studies like this one help identify underlying processes of successful translation in health research and, in so doing, contribute to a more robust science of translation.
